# Hypothermia due to Antipsychotic Medication: A Systematic Review

**DOI:** 10.3389/fpsyt.2017.00165

**Published:** 2017-09-07

**Authors:** Cherryl Zonnenberg, Jolien M. Bueno-de-Mesquita, Dharmindredew Ramlal, Jan Dirk Blom

**Affiliations:** ^1^Parnassia Psychiatric Institute, The Hague, Netherlands; ^2^Faculty of Social Sciences, Leiden University, Leiden, Netherlands; ^3^Department of Psychiatry, University of Groningen, Groningen, Netherlands

**Keywords:** body temperature, neuroleptic, old age, side effect, thermoregulation

## Abstract

**Background:**

Hypothermia is a rare, but potentially fatal adverse effect of antipsychotic drug (APD) use. Although the opposite condition, *hyper*thermia, has been researched extensively in the context of the malignant antipsychotic syndrome, little is known about *hypo*thermia due to APDs.

**Objective:**

This study aimed to review the literature on hypothermia in the context of APD use, and formulate implications for research and clinical care.

**Methods:**

A systematic search was made in PubMed and Ovid Medline.

**Results:**

The literature search yielded 433 articles, including 57 original case descriptions of hypothermia developed during APD use with non-toxic plasma levels. All cases together indicate that the risk of developing hypothermia is highest during the 7 days following initiation, or increase in dosage, of APDs, especially in the presence of additional predisposing factors, such as advanced age, exposure to cold, adjuvant use of benzodiazepines, and (subclinical) hypothyroidism. In addition, data derived from drug-monitoring agencies suggest that the prevalence of APD-related hypothermia is at least 10 times higher than suggested by the literature.

**Conclusion:**

We conclude that health-care professionals need to monitor the body temperature of patients starting with (an increased dose of) APDs for a duration of 7–10 days to prevent hypothermia, especially in the presence of multiple risk factors. Moreover, systematic studies are needed to establish the actual prevalence of APD-related hypothermia as well as the relative risk for individual APDs.

## Introduction

In humans, hypothermia is defined as a core body temperature (or rectal temperature, in clinical practice) of <35.0°C. Traditionally, three degrees of hypothermia are being distinguished, called mild hypothermia (33–35°C), moderate hypothermia (28–33°C), and severe hypothermia (<28°C) (1). After an initial phase of activation, hypothermia causes a process of progressive depression of all organ systems. Thus, mild hypothermia is characterized by heavy shivering, cold diuresis, and a cold, white skin; moderate hypothermia by reduced shivering, hyporeflexia, ataxia, and bradycardia; and severe hypothermia by the cessation of shivering, bradycardia (with possible cardiac arrest), hypotension, hypoventilation (with possible apnea), areflexia, oliguria, coma, and eventually death (2).

As various predisposing factors and underlying mechanisms are known for hypothermia, the condition is conceptualized as having a multifactorial etiology. The term “primary hypothermia” is used when a low body temperature is attributable to environmental factors, including exposure to cold, poor heating, inadequate clothing, and poor nutrition (3). The term “secondary hypothermia” is used when it is attributable to a medical condition, as exemplified by disorders of hypothalamic thermoregulation (4). However, hypothermia has also been reported in the context of the use of antipsychotic drugs (APDs, neuroleptics), including clozapine (5), pipamperone (6), risperidone (7), olanzapine (8), aripiprazole (9), ziprasidone (10), and zotepine (11).

As the causative factors underlying APD-related hypothermia are in need of further elucidation, it remains unclear whether this condition deserves to be classified as a primary or secondary type of hypothermia, or perhaps as a tertiary (i.e., iatrogenic) type. Nevertheless, it is considered a rare, although potentially lethal, adverse effect of APD use, especially in the presence of other predisposing factors for hypothermia, such as advanced age, a cerebrovascular accident, (subclinical) hypothyroidism, sepsis, benzodiazepine use, alcohol intoxication, kidney or liver failure (4, 10), and possibly also poverty (12). To our knowledge, no systematic studies are available on the relative risk contributed by each of these factors, but APD use alone has been held accountable for at least some hypothermia-induced deaths (13).

The aim of this review is to critically assess the risk of hypothermia in the context of regular APD use (i.e., outside the context of toxic plasma levels), to promote the awareness of this potentially lethal adverse effect among health-care professionals, to provide practical advice aimed at preventing and treating it, and to formulate objectives for future research.

## Materials and Methods

For the present review, a systematic search was conducted in the PubMed and Ovid databases (up to May 2017). The Ovid database included EMBASE (1974 through May 2017), Ovid Medline (1946 through May 2017), and PsycINFO (1806 through May 2017). The search terms used were “hypothermia,” “low body temperature,” and “thermoregulation,” which we combined with “antipsychotic” and “neuroleptic.” Included were all relevant papers written in English and Dutch. All cross-references were checked. Excluded were articles on hypothermia in animals and on hypothermia in humans coinciding with toxic plasma levels of APDs. Throughout this paper, we use the term “*additional predisposing factor for hypothermia*” when any factor, additional to the use of APDs, increases the risk of hypothermia even further. The Adverse Drug Reaction (ADR) Probability Scale, also known as the Naranjo Probability Scale (14), was used to assess the causal role of APDs in hypothermia.

## Results

### Literature Search

The literature search yielded 433 articles, of which 94 were considered relevant for the present review. These included 48 case reports, with a total of 63 individual case descriptions. We excluded six cases because: (i) two of them were discussed twice in different publications [i.e., in Ref. (15–18)], (ii) three did not meet the criteria of hypothermia in the context of APD use (19–21), and (iii) one had an incomplete report (22). This resulted in 42 relevant case reports with a total number of 57 original case descriptions (Table 1).

**Table 1:** Cases of antipsychotic-related hypothermia.

Case no.	Reference	Minimum body temperature (°C)	Antipsychotic	Number of additional predisposing factors (characterization)
1	Al Chekakie et al. (15); Ginsberg (16)	33.4	Risperidone	1 (PAH)
2	Blass and Chuen (23)	33.4	Olanzapine	2 (PAH, MD)
3	Brandon Bookstaver and Miller (7)	32.5	Risperidone	3 (PAH, CNS, M)
4	Brevik and Farver (4)	33.3	Risperidone	1 (M)
5	Bueno de Mesquita and Balk (8)	27.0	Olanzapine	3 (PAH)
6	Chen et al. (11)	<35.0	Zotepine	1 (M)
7	Chen et al. (11)	34.8	Zotepine	1 (M)
8	Chen et al. (24)	34.0	Zotepine	0
9	Chu and Nyfort-Hansen (25)	31.9	Olanzapine	4 (PAH, MD, O)
10	Eikenboom et al. (6)	31.8	Pipamperone	2 (PAH, MD)
11	Eikenboom et al. (6)	33.7	Pipamperone	3 (PAH, M, O)
12	Fukunishi et al. (26)	<34.0	Olanzapine	1 (O)
13	Gibbons et al. (10)	29.4	Ziprasidone	1 (PAH)
14	Goodbar et al. (27)	32.8	Thioridazine	4 (CNS, MD, M)
15	Hägg et al. (28)	31.5	Haloperidol	1 (PAH)
			Levomepromazine	
			Olanzapine	
			Thioridazine	
16	Hamuro et al. (29)	32.9	Levomepromazine	1 (PAH)
			Haloperidol	
17	Hamuro et al. (29)	33.0	Levomepromazine	2 (PAH)
			Zotepine	
18	Hamuro et al. (29)	33.0	Sultopride	2 (PAH)
			Haloperidol	
19	Hamuro et al. (29)	34.3	Haloperidol	1 (PAH)
20	Harada et al. (30)	32.0	Haloperidol	3 (PAH, MD, M)
			Chlorpromazine	
21	Hung et al. (31)	34.9	Olanzapine	1 (M)
22	Jepsen and Jestrab (32)	29.9	Risperidone	1 (CNS)
23	Jordan et al. (33)	30.2	Risperidone	1 (PAH)
24	Kamp et al. (34, 35)	31.9	Risperidone	2 (PAH, M)
			Pipamperone	
25	Kamp et al. (34, 35)	34.1	Pipamperone	2 (PAH, M)
26	Kansagra et al. (36)	31.2	Olanzapine	2 (PAH, O)
27	Kreuzer et al. (17)	30.0	Olanzapine	3 (MD, M, O)
28	Kreuzer et al. (17, 18)	31.0	Olanzapine	2 (CNS, M)
29	Kreuzer et al. (17)	33.0	Olanzapine	1 (PAH)
30	Kreuzer et al. (17)	Unknown	Benperidol	1 (CNS)
31	Kreuzer et al. (17)	34.3	Benperidol	1 (M)
			Levomepromazine	
32	Lanska and Harsch (37)	33.3	Thioridazine	4 (PAH, CNS, MD, M)
33	Loughnane (38)	34.7	Chlorpromazine	2 (CNS)
34	MacDonell and Wrenn (39)	32.2	Haloperidol	2 (M, O)
35	MacDonell and Wrenn (39)	33.3	Haloperidol	3 (PAH, CNS, MD)
36	Martinez et al. (40)	29.9	Paliperidone	1 (CNS)
37	Mohan et al. (41)	35.0	Haloperidol	0
38	Noto et al. (42)	<35.0	Zotepine	0
			Fluphenazine	
39	Özyurt et al. (43)	33.3	Clozapine	1 (M)
			Aripiprazole	
40	Papazizis et al. (5)	31.0	Clozapine	1 (PAH)
41	Parris et al. (44)	33.1	Olanzapine	2 (PAH, M)
42	Parris et al. (44)	32.0	Quetiapine	2 (PAH, M)
43	Pelechas et al. (45)	27.2	Haloperidol	3 (PAH, M)
44	Pelechas et al. (45)	27.6	Haloperidol	3 (PAH, CNS, M)
			Clozapine	
45	Perera and Yogaratnam (46)	33.3	Risperidone	1 (PAH)
46	Phan et al. (47)	27.0	Risperidone	3 (CNS, MD)
			Olanzapine	
47	Rasnayake et al. (48)	32.0	Olanzapine	0
48	Razaq and Samma (49)	32.8	Risperidone	2 (PAH)
49	Schwaninger et al. (50)	32.1	Thioridazine	3 (CNS, O)
			Pipamperone	
50	Sethi and Kavarum (51)	30.6	Ziprasidone	0
51	Sharma and Tikare (52)	Unknown	Chlorpromazine	1 (CNS)
52	Signorelli et al. (53)	33.2	Haloperidol	Unknown
53	Signorelli et al. (53)	34.2	Haloperidol	Unknown
54	Signorelli et al. (53)	33.6	Haloperidol	Unknown
55	Van Marum et al. (54)	32.0	Levomepromazine	1 (CNS)
56	Van Marum et al. (54)	29.7	Risperidone	1 (CNS)
57	Young (13)	34.4	Molidone	3 (PAH, CNS)
				Mean: 1.7 (0–4)

*PAH, primary accidental hypothermia; CNS, central nervous system; MD, metabolic disorder; I, infection; M, medication; O, other*.

The mean age of these 57 patients was 55 years (range 0–94), including a newborn who had been exposed *in utero*. The median age was 54 years; 29 patients (51%) were male. The psychiatric diagnosis was known in 53 cases (93%), 26 of them (49%) being schizophrenia spectrum disorder and 6 (11%) bipolar disorder. In 33 patients (58%), hypothermia developed shortly after the initiation of antipsychotic treatment or a dose increase; in 9 of these patients (27%) this happened within 2 days and in 14 (42%) after 2–7 days. Two patients (4%) died, with hypothermia being the most likely cause. For 50 patients (88%), one or more additional predisposing factors for hypothermia (i.e., in addition to the use of APDs) were described in the original papers; in four cases (7%), there were no additional predisposing factors, and in the remaining three cases (5%), no mention was made of such factors. The mean number of additional predisposing factors per patient was 1.7 (range 0–4). The most prevalent ones were advanced age (25 cases, 46%), adjuvant use of benzodiazepines (20 cases, 37%), (subclinical) hypothyroidism (8 cases, 15%), and outdoor exposure to cold (8 cases, 15%) (Table 2).

**Table 2:** Predisposing factors for hypothermia, with examples per category [after Brevik and Farver (4)].

Primary accidental hypothermia	Central nervous system	Metabolic disorders	Infections	Medication	Other
Outdoor exposure to cold	Seizures	Hypoglycemia	Sepsis	Alcohol	Renal failure
Sports-related	Brain injury	Thiamine deficiency		Phenothiazines	Hepatic failure
Inadequate or wet clothing	Brain tumors	Hypothyroidism		Barbiturates	Cardiac failure
Advanced age	Cerebrovascular accident	Hypopituitarism		Benzodiazepines	Starvation or malnutrition
Infants	Hypothalamic disorders	Adrenal insufficiency		Cannabis	Surgery, prolonged
	Parkinson’s disease			Narcotics	Cardiopulmonary resuscitation
	Spinal cord injury			Tricyclic antidepressants	Shock
				Vasodilators: prazosin, terazosin	Burns
				Regional or general anesthetics	Exfoliative dermatologic disorders
				Neuromuscular blockers	Immobility or debilitation

With the aid of the Naranjo Probability Scale (14) we assessed the contribution of each APD to the development of hypothermia. As some patients used multiple types of APD, we recorded 71 instances of APD use for the total number of 57 cases. Table 3 provides an overview of the demographic and clinical characteristics of these 57 cases. The mean outcome of the Naranjo Probability Scale was 1.5 (0–7), with a median of 1, which is indicative of a *possible* ADR. Hypothermia developed most often in the context of the use of olanzapine (13 cases, 18%), haloperidol (13 cases, 18%), and risperidone (10 cases, 14%). However, as indicated by Table 4, hypothermia also developed in association with 14 other types of APD.

**Table 3:** Demographic and clinical characteristics of cases of hypothermia following antipsychotic drug use.

Characteristics	Data from the literature
Number of cases	57
Number of episodes	77
Males	29 (51%)
Age in years: mean (range), median	55 (0–94), 54
	Unknown: 2 (4%)
Number of psychiatric diagnoses	53 (93%)
Schizophrenia/schizoaffective disorder	26 (49%)
Bipolar disorder	6 (11%)
Dementia	6 (11%)
Psychosis NOS	3 (6%)
Delirium	3 (6%)
Mental retardation	4 (8%)
Other	5 (9%)
Pharmacological intervention	
Number of cases of hypothermia after start or dose increase of antipsychotic	33 (58%)
No change	20 (35%)
Unknown	4 (7%)
<2 days	9 (27%)
2–7 days	14 (42%)
>7 days	7 (21%)
Unknown	3 (9%)
Number of deaths	2 (4%)

**Table 4:** Antipsychotics and number of associations with hypothermia.

Type of antipsychotic	Number of associations of hypothermia as found in cases described in the literature (*n* = 57) (%)
Olanzapine	13 (18)
Haloperidol	13 (18)
Risperidone	10 (14)
Levomepromazine	5 (7)
Pipamperone	5 (7)
Zotepine	5 (7)
Thioridazine	4 (6)
Clozapine	3 (4)
Chlorpromazine	3 (4)
Ziprasidone	2 (3)
Benperidol	2 (3)
Sultopride	1 (1)
Fluphenazine	1 (1)
Quetiapine	1 (1)
Paliperidone	1 (1)
Molidone	1 (1)
Aripiprazole	1 (1)
Total	71

*Due to the fact that various patients used more than one antipsychotic drug (APD), the number of associations between antipsychotics and hypothermia is larger than the number of patients. Moreover, as the number of people receiving individual APDs was unknown, the numbers in this table should not be taken as prevalence figures or relative risks*.

In all, 14 patients experienced more than one hypothermic episode (Tables 1 and 2). In these cases, the prescribed APD was nevertheless continued or reintroduced, after which some patients experienced yet another episode. Three patients experienced a second hypothermic episode after they had switched to a different APD. Nevertheless, Chen et al. (11), Kamp et al. (34, 35), and Goodbar et al. (27) describe four cases in which hypothermia did not recur after lowering of the dose, while Özyurt et al. (43) describe a case in which hypothermia did not recur after *increasing* the dose after an interval of 2 days.

## Discussion

### Historical Perspective

The potential effects of APDs on body temperature have been known since the discovery of chlorpromazine by Charpentier in 1950. During the early 1950s, when the compound was yet to be marketed as an “antipsychotic,” it was used during surgery to suppress the body’s response to cooling (55, 56). In a similar vein, Ferguson et al. (57) describe the case of a 69-year-old female who suffered from spontaneous, recurrent hypothermia for years on end, probably due to an agenesis of the corpus callosum, who was treated successfully with the aid of 25–50 mg of chlorpromazine. As an aside, it is noteworthy that during the 1950s hypothermia was investigated as a potential treatment method for schizophrenia (58). Interestingly, Chong and Castle (59) later found that individuals diagnosed with schizophrenia have different baseline temperatures to begin with, although in their study the role of APD use was identified as a possible confounder. Heh et al. (60) found that haloperidol and clozapine both have the potential to lower the body temperature compared to baseline (measured orally in their study), while Shiloh et al. (61) found that the initiation of APD use in male, medication-naive patients diagnosed with schizophrenia is associated with a persistent decrease in body temperature, albeit within the normal range. Likewise, Kudoh et al. (62) found that patients diagnosed with schizophrenia who use APDs have a lower body temperature during surgery than controls.

Antipsychotic drug-related hypothermia was first described by Loughnane (38) in a patient treated with chlorpromazine, and APD-related *hyperthermia* 8 years before that by Delay et al. (63). Today hyperthermia is the better known adverse effect, as it features prominently in the malignant antipsychotic syndrome (MAS, formerly known as malignant neuroleptic syndrome). This other potentially lethal failure of thermoregulation due to APDs has been researched extensively (64). For unknown reasons, hypothermia has received less attention (29, 65), even though a search by van Marum et al. (54) in the *World Health Organization’s Adverse Drug Reaction Database* indicated that an almost equal number of reports had been filed on hypothermia as on hyperthermia in relation to APD use (480 versus 524 cases, respectively). An important reason for this inequality may be that—notably mild—hypothermia tends to present with rather subtle and atypical symptoms, such as confusion, vertigo, nausea, hunger, chills, pruritus, and dyspnea (4) and may, therefore, easily go unnoticed. Moreover, as measurement of the body temperature of patients using APDs is not standard practice and health professionals and researchers may not be familiar with all predisposing factors (Table 2), in clinical practice, many cases may have been missed and, therefore, the problem underestimated.

### Physiology

Maintaining a stable core temperature is a major homeostatic function critical to survival. Under normal circumstances, the human body functions optimally at a core temperature of 36.4–37.5°C (66). To our knowledge, the prevalence of hypothermia in the general population has never been studied systematically. The normal range for men (measured rectally) is 36.7–37.5°C, and for women 36.8–37.1°C. Measured tympanically, it is 35.5–37.5°C for men and 35.7–37.5°C for women (67). Normal thermoregulation involves maintaining a dynamic balance between heat production, heat conservation, and heat loss (68). This complex and intruiging process is not fully understood, but involves at least thermal sensation, hypothalamic integration, and various central feedback mechanisms (69). The general idea is that thermoregulation is centrally coordinated by the hypothalamus, on the basis of feedback from the skin’s cold and heat receptors (1). Heat conservation is promoted by peripheral vasoconstriction and behavioral responses. Heat production is promoted by shivering as well as non-shivering thermogenesis, the latter via increases in levels of thyroxine and epinephrine (70). The hypothalamus has the role of integrating the autonomic, endocrine, and motor systems involved, including the body’s behavioral responses (69). Although the body’s core temperature may fluctuate slightly in a diurnal fashion, it is normally maintained within a relatively narrow range of 0.2–0.5°C, mainly by virtue of adjustments of skin vasomotor responses. Only larger downward fluctuations tend to result in a shivering response (1, 71). The most important brain regions for thermoregulation, as currently known, are the medial preoptic/anterior hypothalamic area (POA), the dorsomedial nucleus of the hypothalamus, the periadequeductal gray matter of the midbrain, and the nucleus raphe pallidus in the medulla, of which the POA is the most thermosensitive area. The neurotransmitters dopamine, norepinephrine, and serotonin play important roles in this process, together with the corresponding receptors upon which they act, i.e., the D1, D2, alpha-1, 5-HT-1, and 5-HT-2 receptors (69).

### Pathophysiology

In the literature on human body temperature regulation, hypothermia is attributed either to heat loss, abnormalities of heat conservation or production, failures of central thermoregulation, or a combination thereof (72). Brevik and Farver (4) distinguish six main etiological groups of hypothermia, comprising (1) primary accidental hypothermia, (2) central nervous system disorders, (3) metabolic disorders, (4) infections, (5) medications, and (6) other (i.e., not otherwise specified) disorders (Table 2). These etiological groups are thought to have different (albeit partly overlapping) pathophysiological mechanisms. The mechanism underlying outdoor exposure to cold is peripheral heat loss, in (subclinical) hypothyroidism it is the impaired conversion of substrate to thermal energy (and thus the inability to produce sufficient heat), whereas in advanced age and sedation due to benzodiazepine use, the mechanism is the reduction of physical activity and the ability to shiver which, in turn, limits the ability to produce sufficient heat (72, 73). In the context of APD use the exact mechanism is unclear, although it is hypothesized that the inappropriate conservation of heat may (in such cases) be due to peripheral vasodilatation as well as a failure of central thermoregulation (8, 39, 65, 74). It is further hypothesized that the inhibition of peripheral responses to cooling may be due to an antagonism of alpha-adrenergic receptors. This might explain hypothermia in the context of the use of chlorpromazine, risperidone, clozapine, and thioridazine, which all act as alpha-adrenergic receptor antagonists (54). In mice studies, Boshi et al. (75) found that peripheral alpha-adrenoceptors may also be involved in the mediation of APD-induced hypothermia.

Under physiological circumstances, D2-receptors help to keep the body’s core temperature at a constant level, while D1-receptors are believed to play a modulating role. This explains why massive antagonism of the D2-receptor may entail a dramatic increase in body temperature, as is the case in MAS. Similarly, decreases in the body’s core temperature are linked to antagonism of the D1-receptor. As stimulation of the 5-HT-1-receptor results in a decrease of body temperature and stimulation of the 5-HT-2-receptor in an increase, it is hypothesized that *antagonism* of the 5-HT-2-receptor may be the cause of hypothermia in patients who use pipamperone or second-generation APDs, which act on this receptor (8, 18, 54, 74). De Langen-Wouterse and van Grootheest (9), who studied the adverse effects of aripiprazole, proposed that hypothermia coinciding with the latter APD may be mediated by a partial agonistic effect on the 5-HT-1A-receptor.

### Sudden Death

In the literature examined for this review, there were two descriptions of deaths associated with APD-related hypothermia. One involved a 45-year-old male, diagnosed with schizophrenia, who developed hypothermia after the start of benperidol and who died due to circulatory failure and coagulation disorder with infarctions of the central nervous system (17). The second one involved a 67-year-old male, also diagnosed with schizophrenia, who developed moderate hypothermia during risperidone use and died due to cardiac arrest (33). Both deaths occurred within days after the patients had been found to be hypothermic. In a retrospective study of drug and alcohol use in hypothermia- and hyperthermia-related deaths, APDs had been used by 5 out of 46 patients (11%) who had died while being hypothermic. In two of these cases (4%) alcohol had also played a role (76). It is suggested that, in addition to these two cases, a substantial number of unexpected and sudden deaths among psychiatric patients over the past 60 years may also be attributable to APD-induced hypothermia (13). The underlying mechanism may have been ventricular fibrillation and asystole during the final stages of severe hypothermia. In such cases, in the absence of any signs of frostbite, hypothermia might easily have been overlooked as a possible cause of death (13). In a survey of 49 cases of sudden deaths associated with the use of APDs and/or antidepressants, Methonen et al. (77) failed to establish such a causal relation. This may illustrate how difficult it is to come to grips with the subject matter in the absence of a systematic study design. Nonetheless, the authors warn against the potential arrhythmogenic effects of APDs.

Another factor to increase the risk of death in APD-related hypothermia is the tendency of cold victims to withdraw and undress themselves, known in the literature as *paradoxical undressing* and the *hide-and-die syndrome* (78). The hide-and-die syndrome coincides with 20% of cases of lethal hypothermia, based on the observation of forensic experts that the bodies of people who die of hypothermia are sometimes found in hidden places (e.g., under a bed or behind a wardrobe). This striking behavior in the face of imminent death is attributed to a primitive reaction pattern, possibly triggered and controlled by the brain stem (78). The term “paradoxical undressing” is used when people who experience hypothermia are found partly or completely undressed. The mechanism underlying this behavior is not fully understood, but may be related to a thermal illusion during severe hypothermia, brought about either by reflex vasoconstriction and an ensuing paralysis of the vasomotor center, or cold-induced paralysis of the nerves in the vessel walls which, in turn, leads to vasodilatation and a paradoxical sensation of heat (78).

### Clinical Practice

In clinical practice, the management of APD-related hypothermia starts with prevention. Therefore, Eikenboom et al. (6) advise to think twice before prescribing APDs, especially in elderly patients. Chahine et al. (79) agree with this, and moreover, advise to stay with atypical APDs because of the alleged lower risk of hypothermia. This is at odds, however, with the finding by van Marum et al. (54) in that 55% of the cases of hypothermia occur in the context of *atypical* APD use (which was found to be 52% in our own analysis). Considering the role of the D1- and 5-HT-2-receptor in thermoregulation, this should perhaps not come as a surprise. All in all, warnings such as those above should not distract us from the fact that, as far as we know, hypothermia may affect anyone using any type of APD, including atypical ones, under widely varying circumstances. This is further indicated by reports such as those by Hung et al. (31), who described hypothermia in an adolescent, by Mohan et al. (41), who described it in a newborn whose mother was treated with haloperidol during pregnancy, and by MacDonell and Wrenn (39), who described two cases that occurred during summer, in a warm environment, in the presence of two, respectively three, additional predisposing factors. Although there is currently insufficient evidence to justify standard screening of the body temperature in all patients receiving a prescription for an APD, individuals at risk deserve to be identified and subsequently monitored.

Van Marum et al. (54) found that 80% of the cases of APD-related hypothermia set in shortly after the start of medication or a dose increase, and, in 73% of these cases, within the first 7 days. This is roughly comparable to the results of our own analysis, which indicates that 58% of the cases set in after initiation or dose increase, 69% of which occurred during the first 7 days. In sum, the first week after APD prescription or dose increase would seem to be the most vulnerable episode. To prevent APD-related hypothermia without unnecessarily burdening both patient and staff, it seems safe to limit temperature measurements to a period of 7–10 days after any initiation or dose increase of APDs. This can be done in several ways and with the use of several devices.

In biomedicine, oral, axillary, and rectal measurements are made with the aid of mercury-in-glass thermometers (although they are now banned in most European countries), tympanic measurements with infrared thermometers, and intra-arterial measurements with needle thermocouples. For most clinical purposes, rectal and tympanic measurements are considered sufficiently reliable, although for accurate results it is mandatory to use a thermometer specifically tested and validated for temperatures below 35°C (2). Meanwhile, the presence of any additional predisposing factors should be assessed. In proven cases of hypothermia, an electrocardiogram is advisable to detect the presence of bradycardia and/or Osborn waves, which are characteristic (although not pathognomonic) of hypothermia (80). Blood tests are then necessary to screen for acidosis, thrombocytopenia, and other coagulopathies, and to assess kidney as well as liver function (2).

### Treatment

The symptomatic treatment of hypothermia involves gradual rewarming, during and after which the patient’s vital signs are strictly monitored (2). Mild hypothermia is best treated by passive external rewarming, e.g., with blankets or other types of isolation material. Moderate hypothermia, as well as mild hypothermia resistant to passive rewarming, is best treated with active external rewarming, e.g., with warm blankets. Severe hypothermia requires treatment on an ICU where warm intravenous fluids can be administered and continuous monitoring of vital functions is possible. Comorbid hypotension is best treated with volume resuscitation and, in therapy-resistant cases, with low doses of inotropic drugs such as dopamine. In this context, bradycardia is usually unresponsive to atropine, but the heart rate tends to restore during rewarming, as do blood pressure and coagulation disorders, if present (2). Thus treated, most cases of APD-related hypothermia tend to resolve within 24–48 h, although Kansagra et al. (36) and Chen et al. (24) both described a patient who needed 9 days. In such cases, it might seem obvious to interrupt or cease the administration of APDs; however, the question whether this is necessary is not easily answered in the face of the current evidence. As we saw, hypothermia has been described in the context of at least 17 different types of APD, while in the case series of 57 patients described above, only 14 patients experienced more than one hypothermic episode, three of which, moreover, after switching to a different type of APD (Tables 1 and 2). Therefore, only in the face of overwhelming evidence that additional risk factors did not play a major role in causing hypothermia, we do recommend to stop the APD currently used during rewarming. When additional risk factors are present, or stopping the APD is not possible, the original treatment can be continued under strict monitoring of the temperature and other vital functions. What may be considered then, instead, is lowering the dose. After normalization of the temperature, there seems to be no strict contraindication for reintroducing the same APD or readministering it at its initial dose. What warrants proper attention, however, is the (continuing) presence of additional risk factors for hypothermia.

## Conclusion

With 57 reports, the number of published cases on APD-related hypothermia is small, especially considering the widespread use of these types of medication worldwide. However, according to drug-monitoring databases, this potentially lethal complication of APD use is at least 10 times that suggested by published reports. Therefore, health professionals need to be aware of hypothermia as a possible adverse effect of APDs, especially in patients with additional predisposing factors for hypothermia who are starting with an APD or whose dose is being increased. Strict monitoring of the body temperature for a duration of 7–10 days is a simple and effective way to prevent complications. Meanwhile, systematic research is needed to shed more light on the issue of thermoregulation in patients diagnosed with schizophrenia, and on the relative risk of all types of APD for mediating hypothermia, whether or not in the presence of any other predisposing factors.

### Limitations

The literature on APD-related hypothermia is limited, especially compared with that on APD-related hyperthermia. Due to the lack of systematic studies, the actual prevalence of this adverse effect is as yet unknown, as is the relative risk for hypothermia for each individual APD and chemical class of APDs in the context of the number of people receiving them. Nevertheless, based on the literature and the above analysis, hypothermia is considered a tangible, and potentially lethal adverse effect of APDs; that said, however, the finding that this is especially true in the presence of other predisposing factors for hypothermia, begs the question of whether these predisposing factors alone might not be sufficient cause for the body temperature to drop. After all, the prevalence of idiopathic hypothermia in the general population is as yet unknown. In other words, a skeptic’s point of view might be that the role of APDs in mediating hypothermia may as well be under- as overestimated. However, the suspicion that APD-related hypothermia is not a naturally occurring disorder, but rather a (potentially fatal) iatrogenic condition, warrants further study and ongoing efforts to prevent and treat it in clinical practice.

## Author Contributions

CZ contributed to the conception and design of the work and to the acquisition, analysis, and interpretation of data for the work, drafted and revised the work, gave final approval for the final version to be published, and agreed to be accountable for all aspects of the work in ensuring that questions related to the accuracy or integrity of any part of the work are appropriately investigated and resolved. JB-d-M contributed to the conception and design of the work, and to the acquisition, analysis, and interpretation of data for the work, revised the work, gave final approval for the final version to be published, and agreed to be accountable for all aspects of the work in ensuring that questions related to the accuracy or integrity of any part of the work are appropriately investigated and resolved. DR contributed to the interpretation of data for the work, revised the work, gave final approval for the final version to be published, and agreed to be accountable for all aspects of the work in ensuring that questions related to the accuracy or integrity of any part of the work are appropriately investigated and resolved. JDB contributed to the conception and design of the work, and to the analysis and interpretation of data for the work, drafted and revised the work, gave final approval for the final version to be published, and agreed to be accountable for all aspects of the work in ensuring that questions related to the accuracy or integrity of any part of the work are appropriately investigated and resolved.

## Conflict of Interest Statement

The research was conducted in the absence of any commercial or financial relationships that could be construed as a potential conflict of interest.
